# Human immunoglobulins are transported to HCMV viral envelope by viral Fc gamma receptors-dependent and independent mechanisms

**DOI:** 10.3389/fmicb.2022.1106401

**Published:** 2023-01-16

**Authors:** Giacomo Vezzani, Silvia Pimazzoni, Rossella Ferranti, Stefano Calò, Giuseppina Monda, Diego Amendola, Elisabetta Frigimelica, Domenico Maione, Mirko Cortese, Marcello Merola

**Affiliations:** ^1^GSK, Siena, Italy; ^2^Department of Environmental Biological and Pharmaceutical Sciences and Technologies, University of Campania “Luigi Vanvitelli”, Caserta, Italy; ^3^Department of Biology, University of Naples Federico II, Naples, Italy

**Keywords:** human cytomegalovirus, viral Fc**γ**-binding proteins, gp68 (*UL119*), gp34 (*RL11*), gp95 (*RL12*), HCMV neutralizing antibodies, human IgGs

## Abstract

Human cytomegaloviruses (HCMVs) employ many different mechanisms to escape and subvert the host immune system, including expression of the viral IgG Fcγ receptors (vFcγRs) *RL11* (gp34), *RL12* (gp95), *RL13* (gpRL13), and *UL119* (gp68) gene products. The role of vFcγRs in HCMV pathogenesis has been reported to operate in infected cells by interfering with IgG-mediated effector functions. We found that gp34 and gp68 are envelope proteins that bind and internalize human IgGs on the surface of infected cells. Internalized IgGs are then transported on the envelope of viral particles in a vFcR-dependent mechanism. This mechanism is also responsible for the incorporation on the virions of the anti-gH neutralizing antibody MSL-109. Intriguingly, we show that gp68 is responsible for MSL-109 incorporation, but it is dispensable for other anti-HCMV antibodies that do not need this function to be transported on mature virions. HCMV-infected cells grown in presence of anti-HCMV monoclonal antibodies generate a viral progeny still infective and possible to be neutralized. This is the first example of a virus carrying neutralizing IgGs on its surface and their possible role is discussed.

## Introduction

Infection by human cytomegalovirus (HCMV) occurs in the majority of the population. Following primary infection, the virus establishes a lifelong latent infection persisting in precursors of dendritic and myeloid cells (Hahn et al., 1998; Forte et al., 2020). Reactivation or infection during immunosuppressive conditions is associated with a variety of diseases (Britt, 2008).

Among *Herpesviridae*, HCMV owns the largest genome and carries several gene products able to counteract host immune responses including viral Fcγ receptors (vFcγRs; Engel and Angulo, 2012; Berry et al., 2020). Human Fc receptors (hFcRs) are present on the surface of most cells of myeloid and lymphoid origin and represent the connection between cell-mediated and humoral immune responses (Bournazos et al., 2016). Immune cells benefit from these plasma membrane molecules as sensors of antigens or immune complexes in the extracellular environment and allow these cells to respond to infection following ligand engagement. IgG production in response to viral infection is a crucial step in activating host effector functions, including neutralization/phagocytosis processes and antibody-dependent cellular cytotoxicity (ADCC; Nimmerjahn and Ravetch, 2008). Several herpesviruses code for viral Fcγ receptors (vFcγRs) that bind the constant region of immunoglobulins. Most of them recognize the constant fragment of IgG (Fcγ) and interfere with the host Fc receptors (Corrales-Aguilar et al., 2014; Jenks et al., 2019; Kolb et al., 2019). Nonetheless, passive transfer of anti-HCMV IgG has been shown to limit viral infection in particular settings, such as upon transplantation, and is used as a therapeutic agent to cure acute cytomegalovirus infection in human patients (Sawyer, 2000; Snydman, 2001). Two putative mechanisms of action have been proposed for the capturing of IgGs by vFcγRs: (a) counteracting neutralizing antibodies *via* a mechanism known as “antibody bipolar bridging” (Frank and Friedman, 1989; Van Vliet et al., 1992; Sprague et al., 2006; Lubinski et al., 2011; Corrales-Aguilar et al., 2014; Ndjamen et al., 2016), a process by which the antibody Fab binds the viral antigen while the vFcR binds the Fc region, impairing its further recognition by immune-system cells and (b) clearing circulating IgGs by capturing them on the surface of infected cells with consequent internalization and lysosome targeting of the antibody (Sprague et al., 2004; Ndjamen et al., 2016). Despite several indications favoring one or the other among these not mutually excluding hypotheses, the functional role of vFcγRs has never been fully elucidated.

Indeed, other herpesviral Fcγ-binding proteins, such as the HSV-1 gE, the murine CMV m138, and the varicella-zoster virus (VZV) gE, are known to play crucial roles in *in vivo* infection independently of their antibody-binding ability (Moffat et al., 2004; Polcicova et al., 2005; Wang et al., 2005; Arapovic et al., 2009; Zerboni et al., 2011). More recently, gp68 and gp34 expressed on infected or transfected cells have been found to antagonize with Fcγ receptors activation forming, according to the antibody bipolar bridging model, ternary complexes including the antigen and the IgG (Corrales-Aguilar et al., 2014; Kolb et al., 2021). Such function has also been described for the product of the *Rh05* gene of the rhesus CMV, where an Fcγ-binding protein belonging to the RL11 family has recently been discovered (Kolb et al., 2019).

HCMV encodes four proteins possessing Fcγ-binding ability (Lilley et al., 2001; Atalay et al., 2002; Cortese et al., 2012). Flow cytometry analysis was used to assess plasma membrane localization and Fc-binding ability of gp34 (gene products *RL11*) and gp68 (gene product *UL119*) expressed from adenovirus vectors as well as transfected gp95 (gene product *RL12*) and gpRL13 (Atalay et al., 2002; Cortese et al., 2012). Binding specificity for human IgG subclasses differs among these proteins. gp34 and gp68 bind all human IgGs subclasses, whereas gp95 and gpRL13 do not bind IgG3 and IgG4 (Atalay et al., 2002; Cortese et al., 2012). The presence on purified viral particles has so far been reported for gp68 by proteomic analysis and for gpRL13 identified as an envelope glycoprotein (Varnum et al., 2004; Stanton et al., 2010; Reyda et al., 2014).

Among the HCMV Fcγ-binding proteins, gpRL13 and gp68 have been reported to internalize following Fc binding at plasma membrane (Cortese et al., 2012; Ndjamen et al., 2016). Endocytosis of viral Fcγ-binding proteins has been well documented for alphaherpesviruses. The VZV gE protein undergoes clathrin-dependent endocytosis mediated by a tyrosine-based motif of its cytoplasmic tail (Olson and Grose, 1997). For pseudorabies virus (PrV), gE is responsible for the ligand-independent internalization of gE:gI since gI alone is unable to undergo endocytosis (Tirabassi and Enquist, 1998). Like its VZV counterpart, the HSV gE C-terminal includes a tyrosine-containing motif crucial for trans-Golgi network (TGN) sorting of the gE:gI complex, an event that also drives correct targeting to the viral particle (Olson and Grose, 1997; Alconada et al., 1999; Wisner et al., 2000). Post-endocytic events include recycling to the plasma membrane or TGN sorting (Alconada et al., 1999). However, gE:gI complex from HSV displays an Fcγ-binding-independent crucial function in cell-to-cell spreading of the virus and requires primary TGN accumulation followed by redistribution to cell–cell junction (Farnsworth and Johnson, 2006).

Intriguingly, it has been shown that anti-gH monoclonal antibody MSL-109 can be uptaken by the infected cells and incorporated on the virion envelope. The viral progeny resulted fully infectious but MSL-109 resistant. Although the involvement of any vFcγRs was not explored, viruses carrying the MSL-109 were inhibited by anti-human antibodies targeting the Fc moiety (Manley et al., 2011).

Here we show that gp34 and gp68, but not gp95, are envelope glycoproteins. In addition, gp68 carries human IgGs and the anti-gH neutralizing antibody MSL-109 on virions. These vFcγ-binding proteins acquire IgGs from the medium and mediate their internalization and insertion into viral particles. Surprisingly, other than MSL-109, all anti-HCMV antibodies tested against specific viral membrane glycoproteins are inserted in the virion in a vFcγR independent route when provided into the culture media to the infected cells. This highlights a potential novel mechanism of viral escape from the immune response.

## Materials and methods

### Cells, plasmids, and antibodies

HFF-1 [Human (*Homo sapiens*) skin/foreskin normal fibroblasts; SCRC-1041] cells were cultured in Dulbecco’s Modified Eagle Medium (DMEM, ATCC 30–2002) supplemented with 15% fetal bovine heat-inactivated serum (FBS, ATCC 30–2020), 100 I.U./ml penicillin and 100 mg/ml streptomycin (Thermo Fisher, Penicillin–Streptomycin 10,000 U/ml).

Primary antibodies were: mouse mAb to Cytomegalovirus IE1 and IE2 (Abcam, ab53495), mouse mAb to gB (Abcam, ab6499), mouse anti-Cytomegalovirus antibody Alexa Fluor 488 (Sigma, MAB810X), mouse monoclonal anti-human IgG (Abcam, IG266), polyclonal rabbit anti-Cytomegalovirus gL (MyBioSource, MBS1498443), mouse mAb to Cytomegalovirus pp65 (Abcam, ab6503), 6xHis Tag antibody (Invitrogen, MA1-213115), anti-KDDDDK Tag antibody (Invitrogen, MA1-91878), mouse monoclonal antibody anti-GAPDH (SIGMA, G8795-200UL), polyclonal anti-HA produced in rabbit (Abcam, ab137838), polyclonal anti-TGN46 produced in rabbit (Abcam, ab50595), and mouse monoclonal anti-Lamp1 (Abcam, ab289548). Recombinant human monoclonal anti-NHBA 5H2 antibody is described in Giuliani et al. (2018). The Fab sequences of monoclonal antibodies 13H11, 1G2 4i22, and MSL-109 were recovered from literature and, once reconstituted as human IgG1, were produced internally (GSK, Rockville site).

Secondary antibodies used in this study were: Alexa Fluor F(ab)_2_ fragments of 488-, 568-, and 647-conjugated goat anti-mouse (Invitrogen) and HRP-conjugated secondary antibodies from Perkin Elmer. Chrompure DyLight 649-, 549-conjugated human Fc fragments were purchased from Jackson ImmunoResearch.

Human (Merck, AG714) IgG Fc fragments were obtained from vendors.

### Immunofluorescence microscopy

**Figure 1:**
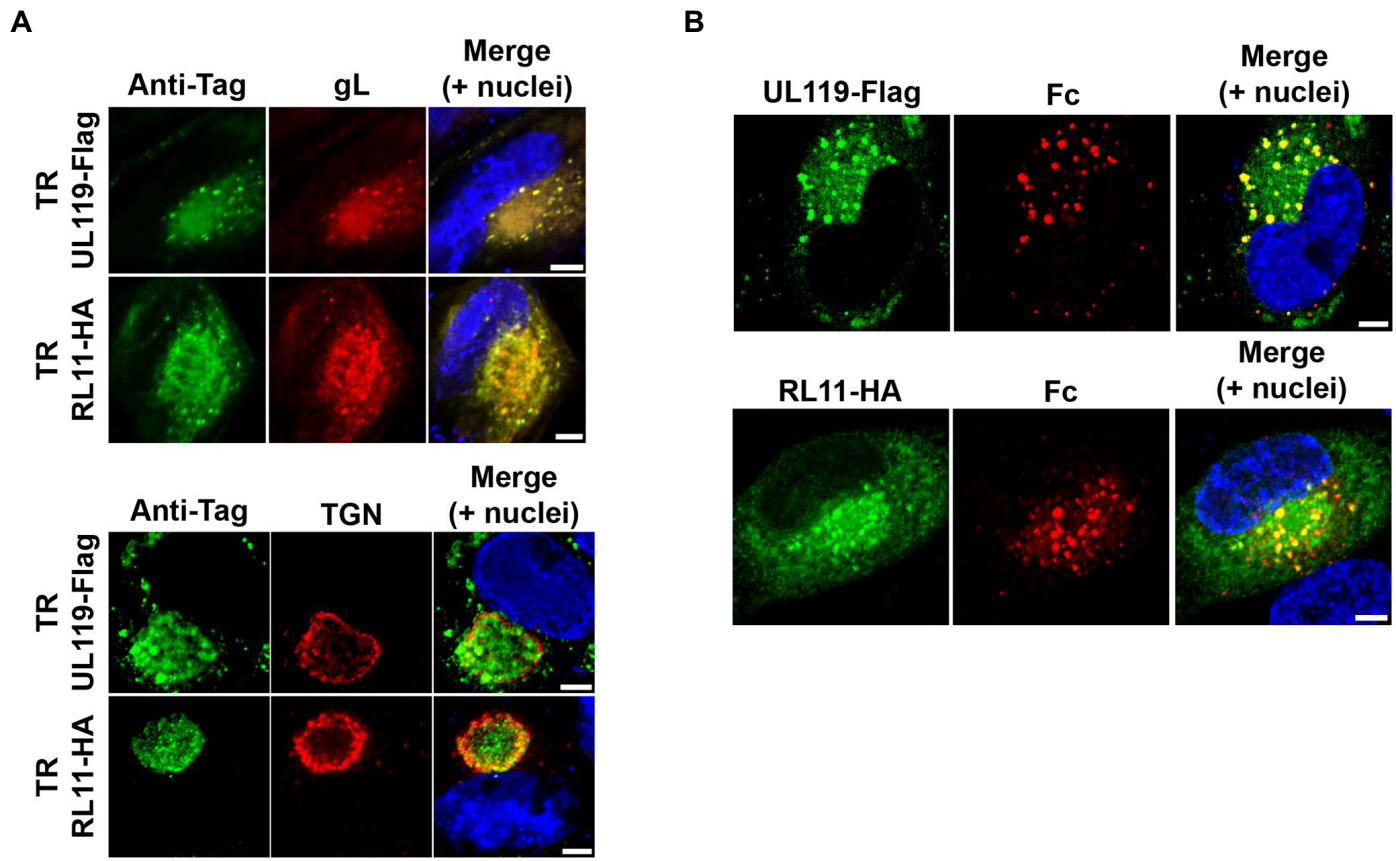
gp68 and gp34 localize within the viral assembly complex in HCMV-infected cells and colocalize with human Fc. **(A)** HFF-1 cells were infected with TR-*UL119*Flag and TR-*RL11*HA (top and bottom rows, of the two panels, respectively). 5 d.p.i. cells were fixed, permeabilized, and stained. *gp68* and *gp34* (green color) were detected with anti-tag antibodies. Envelope glycoprotein gL and trans-Golgi network protein TGN46 were stained with antibodies (red color). Z-stacks were collected with a confocal microscope. Representative single confocal slices of each sample are shown. Scale bars: 5 μm. **(B)** HFF-1 were infected with TR-*UL119*Flag or TR-*RL11*HA. 2 d.p.i. 6.6 μg/ml of 649 Dylight-conjugated human IgG Fc fragment was added to the culture media. After 48 h cells were washed, fixed and stained with anti-tag antibodies (green color). Images were collected through confocal microscopy. Single confocal slices of representative pictures are shown. Superimposition of signals from human Fc (Fc, red color), DAPI stained nuclei (blue color), and anti-tags antibodies are shown in the merge panels. Scale bars: 5 μm.

**Figure 2:**
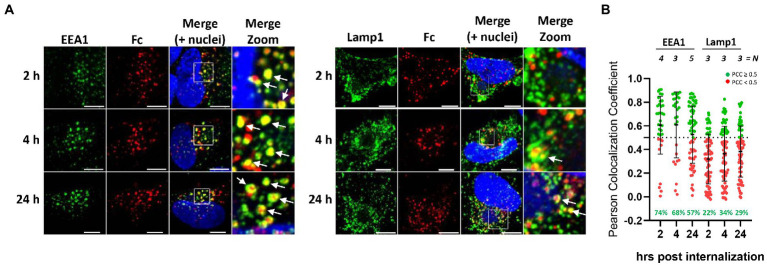
Endocytosed human Fc fragment is retained in the early endosomes. **(A)** HFF-1 were infected with HCMV TR strain. Five d.p.i. cells were incubated with 647 DyLight conjugated human IgG Fc fragment on ice for 60 min before being washed and shifted to 37°C to allow for internalization. At the indicated time points, cells were fixed and stained with antibodies against early endosomes and/or lysosomes (green color, EEA1 and Lamp1, respectively). Images were collected through confocal microscopy. Z-stack projection of representative pictures is shown. Superimposition of signals from human Fc (Fc, red color), cellular markers are shown in the merge panels. Scale bar: 5 μm. White arrows indicate signals overlapping. **(B)** Quantification of data from **(A)**. Z-stacks have been deconvolved and Fc-positive objects have been segmented. For each of them, Pearson’s colocalization coefficients (PCC) between Fc signal and endosomes (EEA1) or lysosomes (Lamp1) was calculated. Each dot corresponds to an Fc-positive object. *N* = number of cells analyzed for each condition. Numbers in green represent the percentage of Fc-positive vesicles colocalizing with the respective cellular marker (Pearson’s coefficient ≥ 0.5).

**Figure 3:**
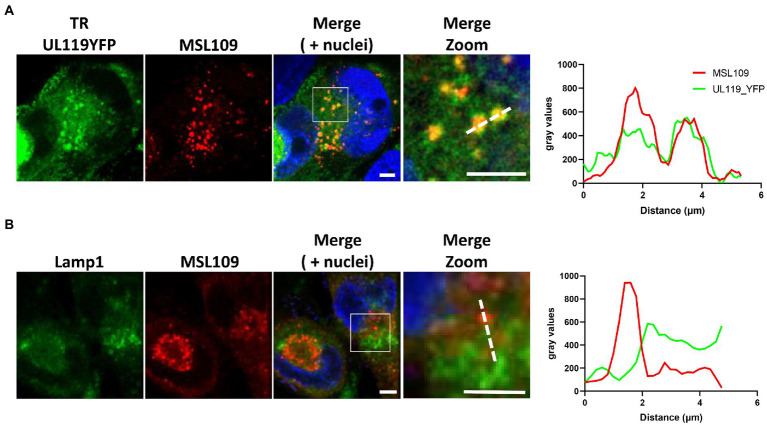
HFF-1 were infected with TR-UL119_YFP and 2 d.p.i incubated with 6.6 μg/ml of human anti-gH antibody MSL-109 for additional 3 days before being fixed, permeabilized, and stained. Secondary antibody Alexa fluor 647 conjugated anti-human was used to detect human antibodies (Red). Lysosomes were stained with anti-Lamp1 antibody. YFP and lamp1 signals are shown in green (**A,B** panels, respectively). Signal intensity profiles along the dotted lines in the zoom panel are shown on the right. Scale bar: 5 μm.

For internalization analyses in infected cells, HFF-1 were seeded on glass coverslips and infected with HCMV at MOI of 1. At different time points post infection (as indicated in the figure legends of Figures 1B, 2, 3), cells were washed in cold PBS and incubated at 4°C with 20 μg/ml of DyLight 649-conjugated human Fcγ for 30 min. After extensive washes, temperature was switched to 37°C to allow for internalization. At the indicated time, cells were fixed with 3.7% paraformaldehyde (Merck, 158127), permeabilized with 0.1% Triton X-100 (Merck, 93418) and incubated in blocking buffer (PBS + 5% FBS) for 30 min before antibody staining. Antibodies were always diluted in blocking buffer. To reduce a specific signal, 5% non-immune human serum was added to blocking buffer. The intracellular locations of antibody-tagged or fluorescent fusion proteins were examined under laser illumination in a Zeiss LMS 710 confocal microscope and images were captured using ZEN software (Carl Zeiss). Image analysis was performed with ImageJ. For colocalization analysis Coloc2 plug-in was used, briefly, a region of interest surrounding individual vesicles was created and the Pearson’s coefficient was calculated. A positive colocalization was assigned if the Pearson’s coefficient was ≥ of 0.5.

### Construction of recombinant HCMV TR strains

Bacterial artificial chromosome (BAC) containing the genome of HCMV TR strain was obtained from Oregon Health and Science University (Murphy et al., 2003). Markerless two-step RED-GAM BAC mutagenesis was used to generate recombinant viruses (Tischer et al., 2010). Briefly, kanamycin resistance cassette, flanked by I-SceI restriction enzyme cleavage site, was PCR amplified from pEP-KanS shuttle vector. The primers used contained homologous regions to allow the integration of the amplicon in the region of interest of the BAC DNA through lambda Red recombinases induction. Combination of I-SceI cleavage with a second Red recombination event removed the resistance gene leaving only the new sequences of interest. Recombination events were performed with *Escherichia coli* GS1783 strain containing a BAC clone of the HCMV TR strain. The *E. coli* strain contains also the lambda Red system and the I-SceI genes under the control of heat shock-inducible and arabinose-inducible promoters, respectively. The desired mutations were confirmed by sequencing of the amplified region and integrity of the whole recombinant HCMV genomes was checked through restriction analysis.

To reconstitute the virus, MRC-5 cells from a confluent T175 cm^2^ flask were trypsin detached, mixed with 10 μl fresh prepared BAC DNA (around 3 μg) and 1 μg of pCMVKm2-pp71 plasmid and electroporated in 4 mm cuvette at 250 V and 950 μF. Supernatant-containing virus was collected from infected cells when cytopathic effects were > 90%.

The following primers were used to generate the tagged versions of *RL11* and *UL119* genes in the TR genome:

for TR-*RL11*HA:

RL11HAFw: 5′-GGATACGGAACCTTTGTTGTTGACGGTGGACGGAGATTTGGAATACCCTTACGACGTGCCTGACTACGCCAGGATGACGACGATAAGTAGGG-3′.

RL11HARv: 5′-TGTCGGTACGTAAGGTTGTTGCGTCTTTGACGGTTGACGCGCATCTTTTAGGCGTAGTCAGGCACGTCGTAAGGGTATTCCAAATCTCCGTCCACCGTCACAACCAATTAACCAATTCTGATTAG 3′ on pEP-KanS plasmid as template.

For TR-*UL119*Flag,

UL119FlagFw: 5′-AGGAGCCCGTTGAGGAAAAGAAACACCCGGTGCCCTACTTCAAGCAGTGGGATTACAAGGATGACGACGATAAGTAGGGATAACAGGGTAAT-3′.

UL119FlagRv: 5′-AAGTCAGCGAAATAAAGACAACACAGCAGCCACTCCTCTCGTCTCGGGCCCTACTTATCGTCGTCATCCTTGTAATCCCACTGCTTGAAGTAGGGCACAACCAATTAACCAATTCTGATTAG-3′ on pEP-KanS plasmid as template.

For the generation of virions missing gp68 (*UL119* gene) expression, the starting codon ATG at position 1 was replaced with a stop codon TGA (TR-*UL119*null). This strategy was required since the *UL119-UL115* region represents a unique transcriptional unit (Leatham et al., 1991) and a gene deletion could have impaired the transcription of the downstream genes. The following primers were used on the UL-*119*Flag-BAC template:

UL119StopFw: 5′-CGCCGTCTCGTCACACGGCAACATGTGTTCCGTGCTGGCGATCGCGCTCGTAGTTGCGCTCTTGGGCGACTAGCACCCGAGAGTGAAAAGTAGCTAGGGATAACAGGGTAATCGAT-3′.

UL119StopRv: 5’-AGTAGACGTGACGGTGGTATTACTAGGGGAAGTGACGGCGCTTGTGGTGCTACTTTTCACTCTCGGGTGCTAGTCGCCCAAGAGCGCAACTACGGCCAGTGTTACAACCAATTAAC-3′.

For the construction of the YFP tagged gp68, first a shuttling plasmid containing the kanamycin resistance cassette within a YFP coding region was constructed. The plasmid was then amplified with oligos annealing at the 3′ of the UL119 gene so that a first Red recombination event would allow the integration of the cassette at the desired site within the CMV genome. The kanamycin cassette was constructed with an I-SceI site at the 5′ end and with duplicated regions of 50 nucleotides upstream and downstream of the kanamycin gene, so that upon I-SceI induction will cleave at the I-SceI site and a second Red recombination event will result in the removal of the cassette leaving an intact YFP sequence.

To insert the kanamycin resistance cassette, a unique BclI site was introduced in the YFP sequence in the pEYFP plasmid *via* quick-change mutagenesis using primers:

*YFP*mutFw: 5′-CAACGAGAAGCGTGATCACATGGTC-3′.

*YFP*mutRv: 5′-GACCATGTGATCACGCTTCTCGTTG-3′.

The kanamycin resistance gene containig the I-SceI site was amplified from the pEP-KanS using the following primers (Bcl1 region underlined, YFP duplicated region in bold):

*YFPKanS*Fw 5′-ATATGATCA**CATGGTCCTGCTGGAGTTCGTGACCGCCGCCGGGATCACTCTCGGCATGG**GATAAGTAGGGATAACAGGGT-3′.

*YFPKanS*Rv: 5′-ATATGATCACAACCAATTAACCAATTCTGATTAG-3′.

The PCR product was cloned in the BclI site within the mutated pEYFP plasmid. Finally, the full cassette was amplified with primers containing the homology regions for the insertion in the UL119 gene upon electroporation of GS1783 *E. coli* strain of the gel purified PCR amplicon and Red mediated recombination as described above:

*UL119YFP*Fw: 5′-AGGAGCCCGTTGAGGAAAAGAAACACCCGGTGCCCTACTTCAAGCAGTGGCGGGATCCACCGGTCGCCACC-3′.

*UL119YFP*Rv: 5′-

AAGTCAGCGAAATAAAGACAACACAGCAGCCACTCCTCTCGTCTCGGGCCTTACTTGTACAGCTCGTCCATG-3′.

Western blot was performed to verify the absence of UL119 expression using anti-FLAG as probe.

The region coding for *RL11*, *RL12,* and *RL13* is an unique mRNA starting from *RL9*, including *RL10,* and terminating at the 3′ end with the *RL13* ORF (Dunn et al., 2003). This region is part of the terminal repeat long (TRL) of the HCMV genomes and in some strains, but not TR, is also present in the internal repeat long (IRL; Murphy et al., 2003). To be sure that translation of the upstream *RL10* gene was not affected, we designed primers used on TR-wt-BAC and TR-*UL119*null that introduced the V5 tag on RL10 gene product (GKPIPNPLLGLDST corresponding to GGCAAGCCCATCCCCAACCCCCTGCTGGGCCTGGACAGCACC DNA sequence) while deleting the *RL11*, *RL12,* and *RL13* genes. In the reconstituted virus, the expression of RL10 was verified by western blot with anti-V5 antibody.

Primers used for generation of TR-Δ*RL11-12-13* were:

Δ*RL11-12-13*Fw: 5′-AACCGACGGGCACAGACGACGAAGAGGACGAGGACGACGACGTCG.

GCAAGCCCATCCCCAACCCCCTGCTGGGCCTGGACAGCACCTAGGGATAACAGGGTAATCGA-3′.

Δ*RL11-12-13*Rv: 5′-GTTAATTGGTTGTAACACTGGCGGCAAGCCCATCCCCAACCCCCTG.

CTGGGCCTGGACAGCACCTGATATGCACATCAATAAACTTTTTTGTATCTTTAGTTATTAATGTCTGTGTG-3′.

All mutants were verified by PCR amplifying the mutated genes and their flanking and by sequencing the amplified regions.

### HCMV virions preparations, fractionation, and Fcs/IgGs incorporation

For the analysis of RL11, RL12, and UL119 localization, mature HCMV virions were separated from dense bodies (DB) and noninfectious enveloped particles (NIEPs) through a positive-density/negative-viscosity step-gradient centrifugation as described previously (Talbot and Almeida, 1977). Briefly, medium from infected cells was collected at 6 dpi or when 90% cytopathic effect was observed and subjected to 600 *g* centrifugation for 20 min at 16°C. The supernatant was transferred to polycarbonate tubes underlined with 20% sucrose and centrifuged for 60 min at 90,000 *g* in a Beckman SW32Ti rotor. Pelleted virus was resuspended in 1 ml PBS and 2 ml of four different solutions containing decreasing concentration of glycerol and increasing concentration of potassium tartrate (from 30% glycerol to 0 and from 10% potassium tartrate to 40%) were layered underneath. Tubes were centrifuged at 317,516 *g* for 60 min at 10°C in a Beckman SW28Ti rotor. The band containing mature virions was collected through a syringe, resuspended in PBS, centrifuged for 60 min at 90,000 *g* in a Beckman SW32Ti rotor, and the pellet-containing virus was resuspended in PBS. Quality of the purification was assessed through negative staining electron microscopy (EM) analysis. To separate envelope from capsid and tegument proteins, purified virions were mixed 1:1 with envelope extraction buffer (1% NP-40, Thermo Fisher 85124, and 4% Sodium Deoxycholate, Thermo Scientific B20759.14) and incubated on ice for 30 min with occasional vortexing. Soluble fraction was collected through max speed centrifugation in a benchtop centrifuge for 30 min at 4°C. The insoluble pellet was washed twice in PBS before being solubilized in SDS-PAGE sample buffer. For each extraction, a total of four confluent T175 cm^2^ flasks of HFF-1 cells were infected. Virus was purified as described above and 10% was mixed with SDS-PAGE loading buffer, while the rest was subjected to protein extraction.

HCMV infection in presence of human Fcγ and IgG was performed according to Manley et al. (2011), with slight modifications. Briefly, 48 h after infection, 6.6 μg/ml of Fcγ or 13.2 μg/ml of IgG were added to the culture medium and incubation prolonged for 2 days. Then, cells were washed and medium was replaced with normal growth medium. Supernatant and infected cell lysates were collected 6 days post infection and cleared by cellular debris by centrifugation at 4,000 × *g* for 20 min. Supernatants were used for cell infection and neutralization experiments or viral particles were isolated on sucrose cushion for western blot analysis. Antibodies neutralization assay was performed by pre-treating viral suspension with 1 μg/ml of the antibody before performing infection of the cells.

### Virus titration By FACS

To titrate viruses, we used a Titration Assay previously described (Britt, 2010) with minor modifications. In brief, 5-fold serial dilutions of samples were performed in DMEM supplemented with 1% fetal bovine heat-inactivated serum and 1 mM sodium pyruvate, and 150 μl of each dilution was applied to duplicate wells of a 96-well flat bottom cluster plate containing 2.5 × 10^4^ HFF-1 fibroblasts, incubated over-night (O/N) at 37°C with 5% CO_2_ before infection. At 24 hpi, the infected cells were trypsinized, transferred in a 96-well round bottom cluster plate, and intracellular staining was performed using a 488 Alexa Fluor conjugated mouse anti-Cytomegalovirus antibody. To detect the fluorescent signal, FACS analysis with BD LRSII Special Order System (Becton Dickinson, San Jose, CA) equipped with High-Throughput Sampler (HTS) option was performed. Titer was calculated using the following equation: Titer (IU/ml) = (N × P)/(V × D) [Note: N = Cell Number in each well used for infection day; P = percentage of fluorescent positive cells (considering the dilution virus exhibiting GFP signal <40%); V = virus volume used for infection in each well (ml); D = dilution fold; IU = infectious unit].

### Viral genomes quantification by digital PCR

Viral genome quantification was achieved through Digital PCR. HCMV infection of HFF-1 cells was performed in DMEM +1% FBS at MOI 1 for 4–6 h before washing and adding fresh DMEM +15% FBS. At the indicated time points, 200 μl of culture supernatant was retrieved, centrifuged at 4,000 × *g* for 10 min to clear cell debris, and treated with DNase (NEB, M0303S 1:100 dilution at 37°C for 1 h). Viral particles were inactivated by 4 min boiling, then samples were stored at −20°C for subsequent analysis. The viral genomes in 1 μl of sample were quantified by Digital PCR performed with QX200 ddPCR EvaGreen Supermix (BIORAD, 186-4036) and the following primers on *UL115* (gL); Fw: ACGCAGGCAGAATTCCTTCA Rv: TAACGTGGTGGTGGCCATAC, following manufacturer’s instructions. Signals were acquired with QX200 Droplet Reader (BIORAD; 186-4003).

### Immunoblotting

Proteins were separated by sodium dodecyl sulfate-polyacrylamide gel electrophoresis (SDS-PAGE) on 4–12% polyacrylamide pre-cast gels (Thermo Fisher, NP0321BOX) under reducing or non-reducing conditions. Proteins (10 μg) were loaded for each cellular lysate. Proteins were transferred to nitrocellulose membranes (iBlot 7-Minute Blotting System, Invitrogen), and membranes were blocked with PBS containing 0.1% Tween 20 (Thermo Fisher, TA-125-TW) and 10% powdered milk (Sigma-Aldrich, M7409). Antibodies were diluted in PBS containing 0.1% Tween 20 and 1% powdered milk. For detection of primary antibody binding, horseradish peroxidase-conjugated anti-rabbit or anti-mouse IgG antibodies and the Chemiluminescent Peroxidase Substrate (Sigma-Aldrich, 34578) were used, according to the manufacturer’s instructions. The densitometric analysis of signal intensity in Western blotting was performed with Image Lab software (BIORAD).

## Results

### Viral Fcγ receptors gp34 and gp68 colocalize with internalized Fcγ in the viral assembly complex (vAC)

Clearance of cell surface-bound human IgG by vFcγRs has been proposed as a method of immune-system recognition escape exploited by HCMV during infection. Based on the observed rapid turnover of gp34 and gp68, Atalay and co-workers suggested their potential involvement as transport device shuttling internalized human IgGs from the plasma membrane into lysosomes (Atalay et al., 2002). More recently, Ndjamen et al. (2016) reached the same conclusions in transfected cells. We decided to analyze internalization of human Fcγ by gp68 and gp34 in the context of the HCMV infection. We constructed two recombinant TR viruses coding for single-tagged species of both viral Fcγ-binding proteins adding Flag and HA tags to the C-terminus of gp68 and gp34, respectively. First, we defined the intracellular distribution of both proteins in human foreskin fibroblast (HFF-1) infected with HCMV TR strains coding for gp68 and gp34 tagged proteins by immunostaining of the infected cells with anti-flag and anti-HA antibodies 5 days post infection followed by confocal analysis. Both proteins were found to accumulate in the perinuclear region, reminiscent of the cytoplasmic vAC, showing almost complete colocalization with the envelope glycoprotein gL and partial colocalization with the trans-Golgi network marker TGN46 (Figure 1A), potentially indicating their localization in the cytoplasmic vAC. Indeed, the TGN46 signal forms a rim surrounding the region of gp68 and gp34 signal, consistent with the reported reorientation of trans-Golgi compartment at the outer edge of the vAC (Das et al., 2007).

To verify the ability of gp68 and gp34 to bind and internalize human Fcγ in our system, we incubated the infected cells with human Fcγ. HFF-1 were infected with TR-UL119Flag or TR-RL11HA at MOI of 1 and, 48 h later, fluorescent human IgG Fc fragment was added to the culture media. Following additional 48 h incubation, cells were washed and treated for confocal microscopy analysis. Anti-Flag and anti-HA antibodies were used to analyze the localization of gp68 and gp34, respectively, and the co-localization with Fcγ in infected cells (Figure 1B). Both gp68 and gp34 showed a vesicular pattern and a more diffuse one (Figure 1B, green), while internalized Fcγ colocalized completely with gp68 and gp34 in vesicles (Figure 1B), suggesting that vesicular inclusion of both viral proteins could be dependent on ligand binding and internalization, consistent with a role for the vFcγRs in the human IgG endocytic activity (Cortese et al., 2012; Ndjamen et al., 2016).

### Internalized Fc fragment accumulates in early endosomes of HCMV-infected cells and only a fraction reaches lysosomes

To investigate further the fate of internalized Fcγ, time course colocalization studies were performed on infected cells. Fluorescent human Fcγ was transiently added to HFF-1 5 days after infection with HCMV strains tagged TR and internalization was followed at different time points using confocal microscopy. A strong overlap between Fcγ fragment and Early Endosome Antigen 1 (EEA1) signals appears as soon as 2 h post internalization and over 50% of total internalized Fcγ fragments remain associated with this compartment at 24 h post-incubation. Conversely, less than 35% of the total Fcγ positive vesicles were co-stained with lysosomal compartments at all time points (Figures 2A, B). Next, we sought to analyze if antibodies directed against viral proteins present on the membrane of HCMV-infected cells follow the same pathway observed for Fcγ fragments upon internalization. To this aim, we generated a mutated TR HCMV virus expressing a YFP fluorescent protein tag at the C-terminus of gp68 and repeated the internalization experiment as above using the human monoclonal anti-gH antibody MSL-109 instead of Fcγ fragment. The internalized MSL-109 was found associated with gp68-YFP signal in perinuclear vesicles and mostly excluded from the lamp1-positive lysosomes (Figures 3A, B, respectively). Taken together, these data suggest that both the internalized Fcγ and anti-viral IgGs remain mainly associated with the gp68 protein and only a fraction of these molecules reaches the lysosome for degradation.

### gp68 and gp34 are envelope glycoproteins

**Figure 4:**
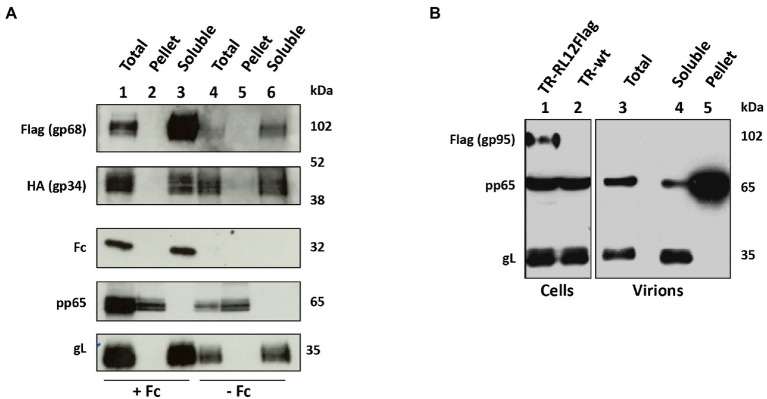
gp68 and gp34, but no gp95, are envelope proteins. **(A)** HFF-1 cells were infected at MOI 1 with either HCMV TR-*UL119*Flag or *RL11*HA in duplicate. Human Fcγ fragments were added to one sample for each virus while the other was grown in normal medium. At day 6 p.i., supernatants were harvested, virions purified through a glycerol/tartrate density gradient and tegument/capsid fraction (Pellet) was separated from envelope proteins (Soluble) through triton X-114 procedure. Lysates of infected cells and fractions of purified virions were analyzed through western blot using antibodies against gL, pp65, human IgG, anti-Flag, or anti-HA. **(B)** HFF-1 cells were infected at MOI 1 with HCMV TR-*RL12*Flag. Infected cells were incubated with human Fcs as described above. At day 6 p.i., supernatants were harvested, and virions were purified through a glycerol/tartrate density gradient. Tegument/capsid fraction (Pellet) was separated from envelope proteins (soluble) through detergent extraction. Lysates of infected cells and fractions of purified virions were analyzed through western blot using indicated antibodies.

Among the four HCMV Fcγ-binding proteins, only gpRL13 is known to be a structural envelope glycoprotein (Stanton et al., 2010). We sought to verify if the vFcγ gp68 and gp34 localized on the viral envelope. In addition, to test if, similarly to what has been reported for MSL-109 (Manley et al., 2011), human IgG fragments could be inserted into the viral particle, Fcγ fragments of human IgG were supplemented into the medium 48 h after infection. Incubation with the supplemented medium was prolonged for 2 days before being changed and replaced with normal growth medium. Supernatant and infected cell lysates were collected 6 days post infection and virions from human Fcγ and untreated cultures were purified from the cell culture medium using negative-viscosity positive-density gradient, before being lysed and subjected to detergent fractionation to separate capsid and tegument fractions. Samples were then treated for western blot analysis and a representative result from these experiments is shown in Figure 4A. Both gp34 and gp68 were revealed to be associated with the lipidic fraction of the virions (Figure 4A), and, thus, are structural proteins exposed on the envelope of mature virions. In addition, the human Fcγ was detected in purified virions, while no bands were observed in the untreated control (Figure 4A). Fc fragment of human IgG was found to co-purify with envelope glycoprotein L (gL) and both vFcγRs gp68 and gp34 in the envelope fraction (lane “soluble” of Figure 4A), while none of these proteins were found in the tegument/capsid fraction (pellet, defined by the presence of tegument protein pp65, Figure 4A). Interestingly, virions purified from cells infected with a recombinant virus carrying a tagged version of *RL12* showed absence of the gp95 signal in the virion-associated fractions (Figure 4B), suggesting that differently from all the other vFcRs, gp95 is a nonstructural protein. This finding highlights potential differential roles for the four vFcRs.

Taken together, our experiments on infected cells and HCMV purified particles are consistent with a role of gp68 and gp34 in the uptake of Fcγ from the plasma membrane and, following transport to the viral assembly complex, insertion into the envelope of nascent virions.

### Fc fragment and human IgG are carried on virions mainly by gp68

To identify which one of the three vFcγRs present on virions was responsible for the envelope transport of Fcγ, three recombinant viruses were generated: (a) TR-*UL119*null that lacks expression of gp68 due to insertion of a stop codon in place of the start codon, (b) TR-Δ*RL11-12-13* that carries deletion of the *RL11*, *RL12,* and *RL13* genes, and (c) TR-*vFcγRs*null that combines the two mutations and abolishes expression of all four vFcRs. A schematic representation of the viruses generated is shown in Supplementary Figure 1A. None of the mutations or their combinations, affected viral replication (Supplementary Figure 1B).

**Figure 5:**
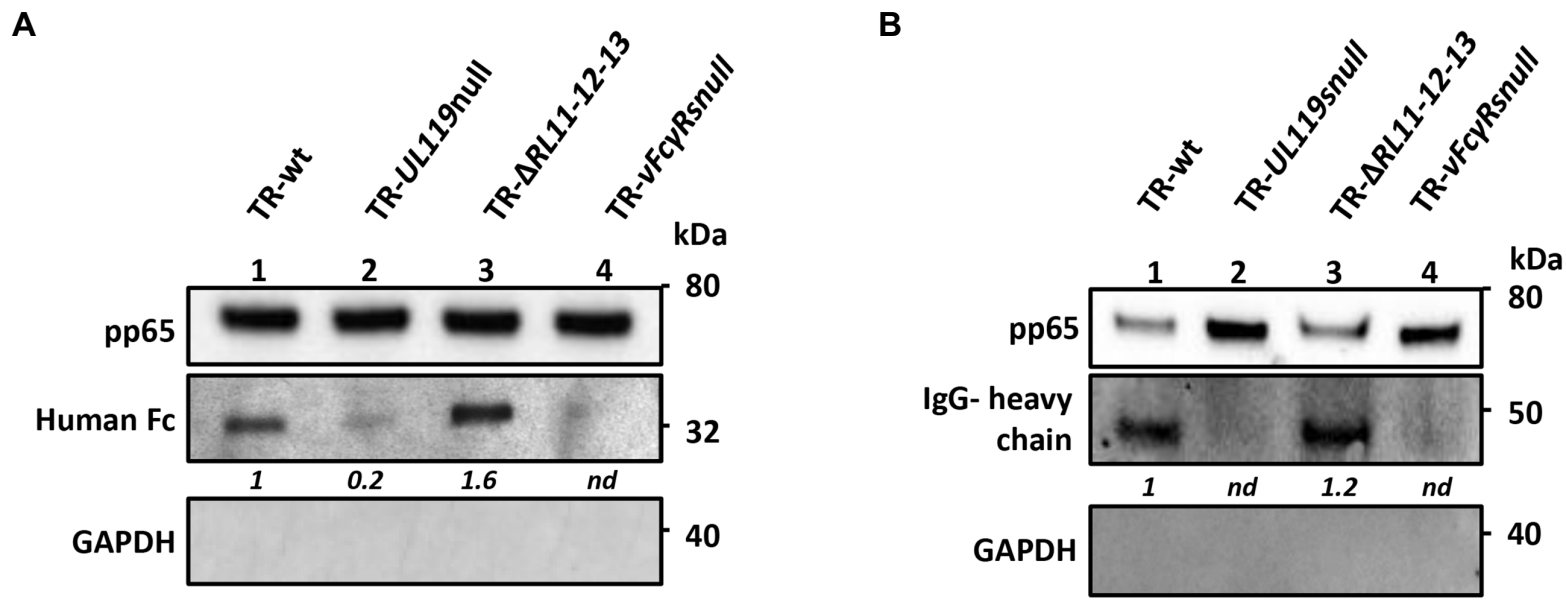
gp68 is the main protein responsible for transporting human Fc and IgGs on virions. HFF-1 cells were infected with HCMV TR-wt, TR-*UL119*-null, TR-Δ*RL11-12-13,* and TR-*vFcγRs*-null mutant. 48 hpi, 6.6 μg/ml of human Fcγ **(A)** or 13.2 μg/ml of recombinant human IgGs **(B)** were added to the culture media. At day 4 pi medium was replaced with fresh DMEM 15% FBS and supernatant was collected at 6 dpi. Viral particles were purified through a sucrose cushion. Pelleted viruses were treated for SDS-PAGE and western blot analysis. Anti-human HRP-conjugated IgG antibody was used to reveal Fcs **(A)** and IgGs **(B)**. Anti-pp65 was used as markers of viral tegument. Anti-GAPDH was used to exclude cellular contaminations.

To identify the vFcR responsible for carrying the human Fcγ to the viral envelope, cells were infected either with wild-type (wt) or with vFcRs deleted viruses at an MOI of 1 and 48 h post infection, 6.6 μg/ml of Fcγ was added to medium. Incubation with the supplemented medium was prolonged for 2 days before it was changed and replaced with normal growth medium. At 6 days post infection, supernatants were used to prepare virions by sucrose cushion. Virions were lysed in detergent-containing buffer and then treated for western blot analysis. Results are shown in Figure 5A. In the absence of gp68, the band corresponding to human Fc was strongly reduced (Figure 5A, lane 2), while it almost disappeared in the mutant missing all 4 vFcRs (Figure 5A, lane 4). On the contrary, deletion of *RL11-R12-RL13* genes did not alter the ability to incorporate human Fc fragments.

Next, we wanted to verify if IgGs could be carried on the virions like the Fc fragments. To this aim, we used a recombinant probe molecule, since commercial preparations of human IgGs are purified from donors not tested for HCMV positivity and could recognize envelope glycoproteins. To avoid the risk of binding through the Fab region of IgG, we added on a human IgG1 scaffold a monoclonal Fab moiety recognizing the *Neisseria meningitidis* protein NHBA (Giuliani et al., 2018). Once expressed and purified, we used this recombinant IgG (rIgGs) to check its binding to vFcγRs and the transport on the virion envelope. HFF-1 cells were infected at an MOI of 1 and 48 h post infection, 13.2 μg/ml of rIgGs was added to medium. The incubation was prolonged for 2 days after which medium was removed and replaced with normal growth medium. At 6 days post infection, virions were purified by sucrose cushion and treated for immunoblotting using anti-human antibodies. A representative immunoblot is shown in Figure 5B. Similarly to Fc, IgG transport on the virions is mainly mediated by gp68 (Figure 5B, lane 2), while contribution of gp34, gp95, and/or gpRL13 appears to be negligible (Figure 5B, lane 4).

Altogether, these experiments reveal that HCMV infection carried out in presence of human IgGs led to viral incorporation of these molecules predominantly *via* gp68.

### Virion localization of anti-HCMV human monoclonal antibodies

Next, we sought to verify if vFcγRs were able to carry anti-HCMV monoclonal antibodies on viral particles. HFF-1 were infected with either TR-*vFcγRs*null or TR-wt and grown in presence of control or anti-HCMV monoclonal antibodies as reported in the previous paragraph. The monoclonals used are as follows: anti-gH 13H11 (Macagno et al., 2010), MSL-109 (Nokta et al., 1994), anti-UL130/131A 4I22 (Macagno et al., 2010), and anti-gB 1G2 (Potzsch et al., 2011). The Fab sequence of each antibody was cloned on a human IgG1 backbone. Except for 4I22, all the selected antibodies are reported to neutralize HCMV infection in HFF-1. The recombinant human anti-NHBA was used as control.

**Figure 6:**
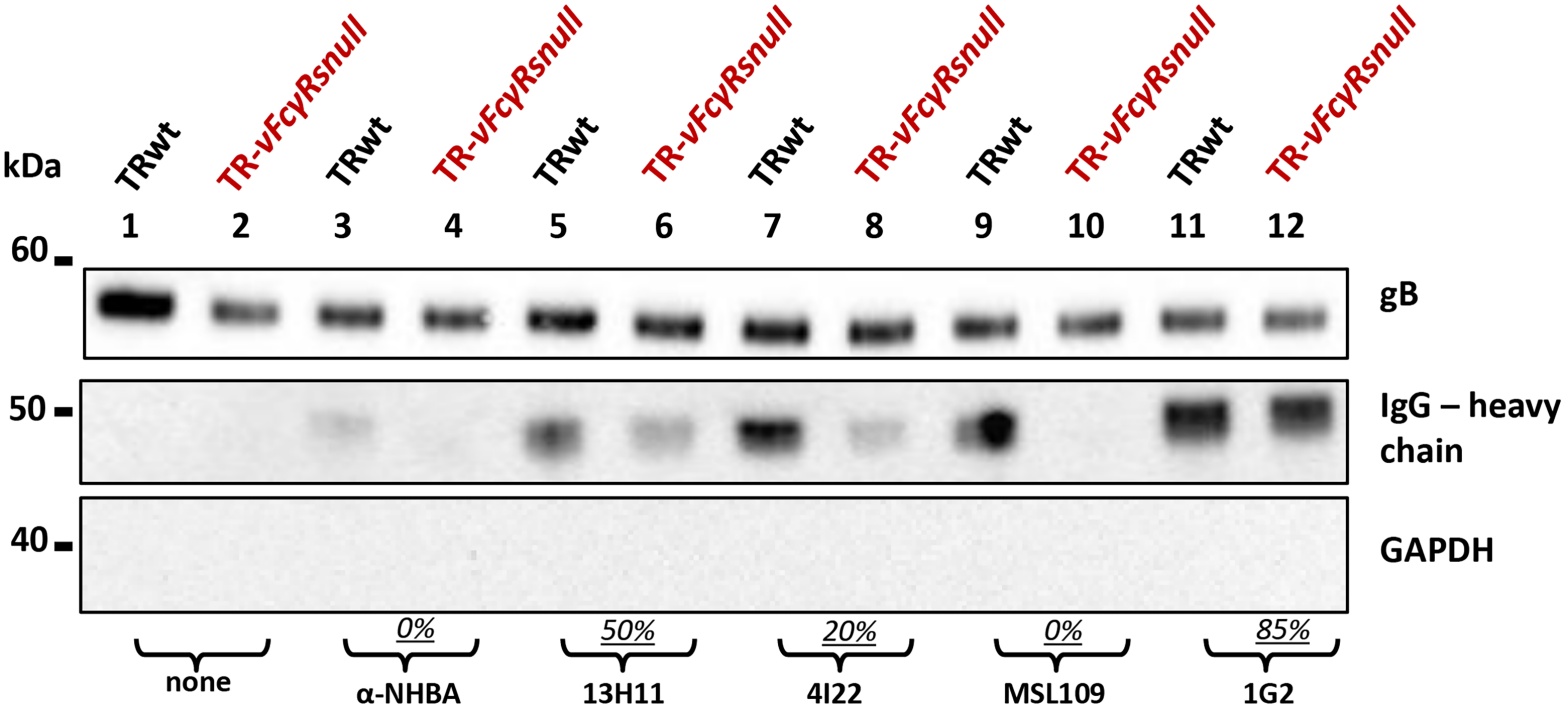
Monoclonal anti HCMV glycoproteins are transported on virions by vFcγRs dependent and independent mechanisms. HFF-1 cells were infected with HCMV TR-wt or TR-*vFcRnull*. At day 2 p.i., human monoclonal antibodies (indicated at the bottom of the figure) were added to the culture media. After incubation at 37°C for 48 h, cells were washed and incubated with fresh medium for additional 48 h. Pelleted viruses were treated with SDS-PAGE and western blot analysis. Human IgG heavy chains (middle panel) were revealed with HRP-conjugated anti-human IgG antibody. Anti-gB (upper panel) and anti-GAPDH (bottom panel) were used as viral protein marker and to exclude cellular contaminations, respectively.

To generate TR-*vFcγRs*null or TR-wt coated with each antibody, we used the procedure detailed above. Virions collected from culture media and purified on sucrose cushion were immunoblotted using HRP-conjugated anti-human as probe. As shown in Figure 6, all monoclonal antibodies were carried on virions but revealed a differential dependency from vFcγRs. The unrelated anti-NHBA antibody and the anti-gH MSL-109 were transported *via* vFcγRs and were completely absent on virions derived from the null mutant (Figure 6, lanes 3, 4 and 9, 10). The anti-gB 1G2 was inserted in virion in a vFcγRs independent manner, as shown by equal band intensity in absence of vFcγRs (Figure 6, lanes 11–12). The anti-gH 13H11 and the anti-UL130/131A 4I22 reached the virion in both ways: in absence of vFcγRs, they were present on virions, but their amount was higher in wt viruses (Figure 6, lanes 5, 6 and 7, 8).

These data suggest that at least two mechanisms might contribute to the transport of human IgGs on the virion envelope, either through binding of the Fc portion by vFcγR, or through binding of the Fab portion to the target viral glycoproteins recognized on the infected cell membrane in case of anti-HCMV specific antibodies.

### Infectivity and neutralization of virions coated with anti-HCMV human IgGs

To explore if envelope-carried antibodies interfere with infectivity and neutralization by anti-HCMV human IgGs, we compared these two parameters in HFF-1 infection of antibodies-coated and uncoated TR-wt and TR-*vFcγRs*null virus mutants.

HFF-1 cells were infected with the TR wt and TR-*vFcγRs*null in duplicate. One plate of each infected cell was incubated with 13.2 μg/ml of each mAb 48 h post infection and allowed to internalize for additional 48 h. The culture media were replaced with fresh medium without antibodies. At day 6 post infection, viruses were recovered on sucrose cushion and resuspended in DMEM 1% FBS medium. Viral genomes were quantified by digital PCR and used at an MOI 1 to infect fresh HFF-1.

**Figure 7:**
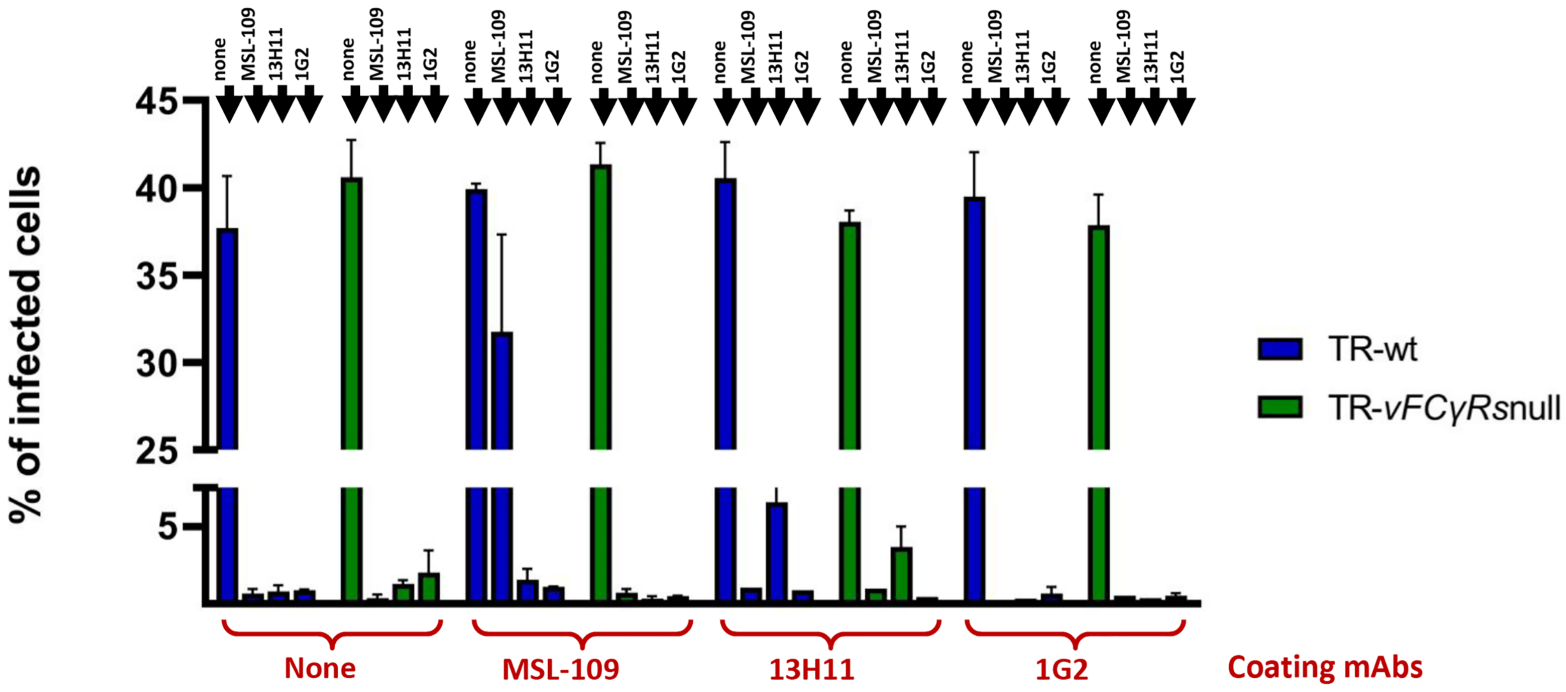
Neutralization assays on untreated and mAbs-coated TRvFcγRs-null and parental strain. HFF-1 were infected with either TR-wt or TR-*vFcγRs*-null at an MOI of 1 and infection carried on for 48 h before adding 13.2 μg/ml of the mAb as indicated in the legend. Medium was changed at day 4 p.i. and replaced with fresh medium. At day 6 p.i., culture media were collected, cleared from cellular debris, and viral genomes quantified by Digital PCR. Fresh HFF-1 were infected at MOI 1 with viruses untreated or pre-incubated with the mAb indicated on the *x*-axis. Antibodies used for neutralization are specified on each bar. Percentage of infected cells was calculated 24 hpi by cytofluorimetry using an anti-CMV 488-conjugated antibody.

Neutralization assays were performed by pre-incubating the viral suspension with anti-HCMV neutralizing antibodies. Results are shown in Figure 7. All mAbs-coated wt viruses resulted infectious as the uncoated controls. In agreement with Manley et al. (2011), MSL-109 inhibits viral entry of the TR-wt virus but does not fully neutralize the MSL-109-coated virus. This protective effect is restricted to MSL-109 coated viruses since viruses coated with 13H11 or 1G2 are still susceptible to MSL-109 neutralization. Conversely, TR-*vFcγRs*null previously incubated with MSL-109 results sensitive to the neutralization activity of MSL-109. This is not unexpected considering that the TR-*vFcγRs*null cannot tether the MSL-109 on its surface (see Figure 6). Additionally, the 13H11 and 1G2, carried on viruses irrespective of vFcR presence (see Figure 6), can neutralize both wt and vFc*γ*Rnull coated and uncoated viruses. Except for MSL-109, coating does not shield against the neutralization activity of antibodies, ruling out a nonspecific mechanism of protection by sheltering of viral particle with human IgGs.

### Antibodies targeting the Fc portion of human IgG are able to neutralize viruses coated with anti-HCMV monoclonal antibodies

**Figure 8:**
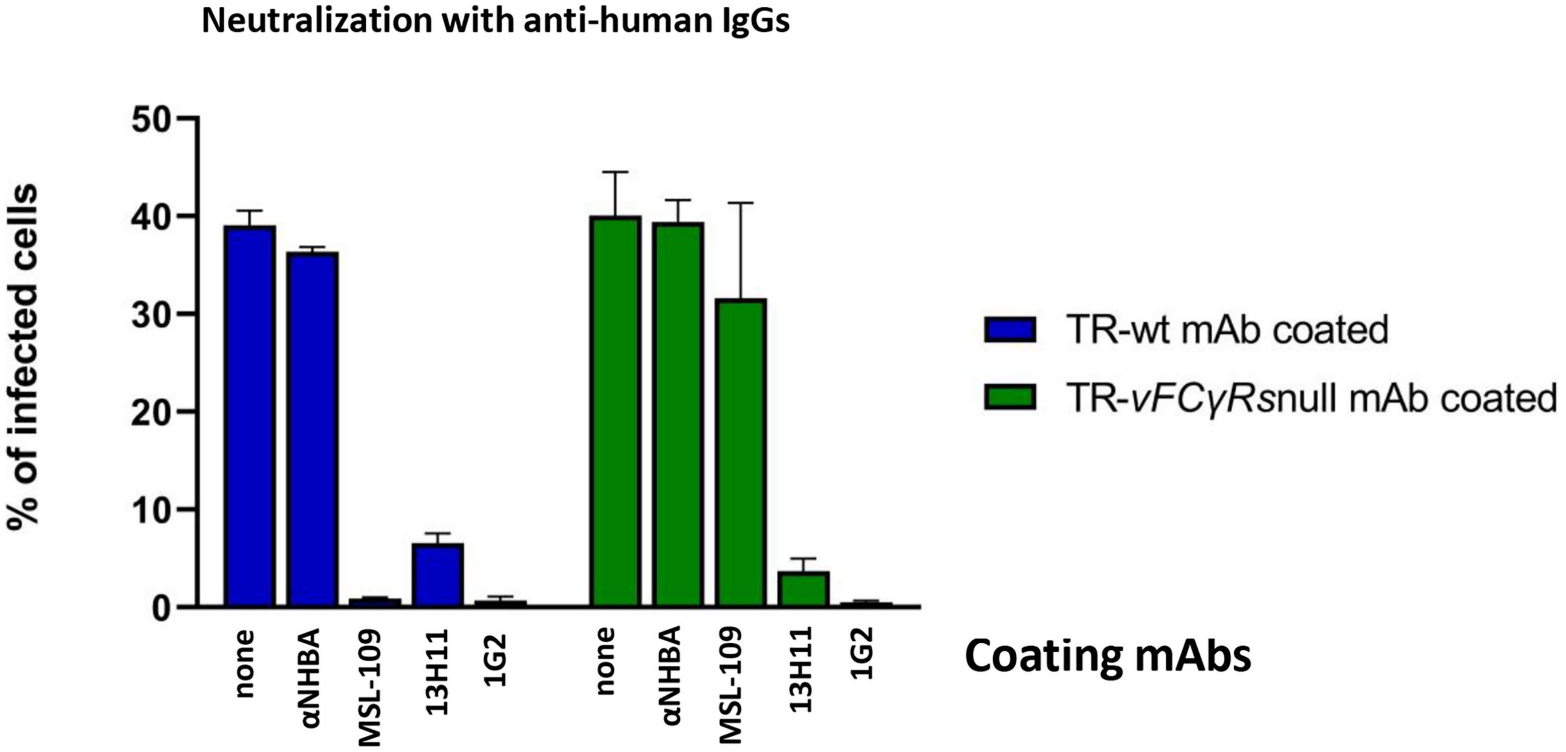
Neutralization assay on mAbs-coated viruses with anti-human antibodies. TR-wt and TR-*vFcγRs*null coated with the mAb indicated on each bar and untreated were prepared as described in legend of Figure 7. Viral genomes were quantified by Digital PCR and fresh HFF-1 infected at MOI 1 with viruses pre-incubated with anti-human antibodies. Percentage of infected cells was calculated by cytofluorimetry using an anti-CMV 488-conjugated antibody.

Manley et al. (2011) reported that virus coated with the MSL-109 antibody could be neutralized by anti-human antibodies. To check if this observation could be extended to different antibodies associated with virions, we repeated the same experiment testing in parallel the 13H11 anti-gH, the 1G2 anti-gB and the NHBA rIgGs coated viruses. Viruses were grown in presence of the mAbs and, following purification, the viral suspensions were incubated with anti-human antibodies before infecting HFF-1. Both wt and v*FcR*null virus were used. The results, presented in Figure 8, show that all mAbs-coated viruses, except anti-NHBA-coated ones, are inhibited in these conditions. For anti-NHBA, a possible explanation could be the low amount of anti-NHBA present on the virions; however, we cannot exclude that the anti-Fc-mediated neutralization requires that the Fab moiety is engaged in binding its specific antigen.

Lack of inhibition is also observed in the context of the v*FcR*null viruses grown in presence of either anti-NHBA and with the MSL-109. However, our previous experiments have highlighted that both immunoglobulins are not carried on viruses lacking vFcR (Figure 6); hence, no binding and neutralization by anti-human IgG is expected in these conditions.

Altogether, these results attest that viral particle carrying human IgGs are not impaired in infectivity irrespective of how antibodies are positioned on the envelope.

Additionally, consistent with Manley et al. (2011), the Fc domain of virus-bound antibodies is important for viral entry since blocking the Fc moiety can inhibit HCMV infection. This effect seems not to be restricted to MSL-109 coated virus but could also be observed to more, if not all, anti-HCMV mAb-coated viruses.

## Discussion

In this study, we have shown the presence of gp34 and gp68 on the envelope of the HCMV and refined previous reports suggesting the presence of these two proteins in viral particles. Indeed, mass spectrometry analysis of purified virions (Varnum et al., 2004; Reyda et al., 2014) revealed the presence of peptides derived from proteolytic digestion of both gp68 and gp34, suggesting a virion association of these glycoproteins. Earlier, Stannard and co-workers identify Fc-binding activity by immunoprecipitation analysis of purified virions associated with a MW of 69 and 33 kDa, sizes compatible with gp68 and gp34. However, by EM studies, they concluded that the two proteins were present within the tegument (Stannard and Hardie, 1991).

Unique among viruses, HCMV codes for four vFcγ receptors as type I transmembrane glycoproteins that during the viral life cycle are exposed on the surface of infected cells. In this localization, gp68 and gp34, the two more extensively studied HCMV vFcγRs, have been found to (i) impair antibody-dependent cellular cytotoxicity *via* a so-called antibody bipolar bridging (ABB) mechanism in which the Fc-mediate capturing of the host antibody linked to a viral glycoprotein and lead to internalization and degradation of the complexes, and (ii) antagonize the activation of cellular Fcγ receptors on cells of the innate immune response (Sprague et al., 2008; Corrales-Aguilar et al., 2014; Ndjamen et al., 2016; Kolb et al., 2021). Still, obscure is the role of the two other FcγRs gpRL13 and gp95. In this report, we have demonstrated that gp95 is a nonstructural protein, while the fourth Fc-binding protein coded by HCMV, gpRL13, was assigned as structural envelope protein by Stanton et al. (2010).

The ABB model dictates that, once bound by the antibody on the plasma membrane *via* the Fc moiety by the vFcγRs, the complex must enter the endocytic pathway. Indeed, uptake of both human IgG and Fcγ fragment in HCMV-infected cells was first reported in 1976 by Keller and co-authors (Keller et al., 1976). Later, Fcγ-binding ability was observed both in the vAC and in the virion tegument (Murayama et al., 1986; Stannard and Hardie, 1991; Alwine, 2012). Cellular Fcγ receptors internalize small immunocomplexes and monomeric IgG on clathrin pits formation and both HCMV vFcγRs gp68 and gp34, when tested for endocytic activity in a transient transfection system, rely on the same internalization mechanism of their cellular counterpart (Hunziker and Fumey, 1994; Matter et al., 1994). In the case of the cellular Fc receptors, the bound immunoglobulin can be either recycled back to the membrane or delivered to the lysosome for degradation. Recycling is often associated with binding to monomeric immunoglobulin and is used by neonatal FcR to increase the serum half-life of circulating IgG or by FcγR to transport immunoglobulins across the epithelial surface (Nimmerjahn and Ravetch, 2008). On the other hand, cross-linked immunoglobulin usually triggers delivery to lysosomes as in the case of Fcγ-bound immunocomplexes (Zhang and Booth, 2010). HSV gE-gI complex is able to bind IgG at neutral pH but no binding was observed at pH 6 (Sprague et al., 2004), supporting a role in antiviral immunocomplex clearance with gE-gI acting as transport device shuttling IgG from the cell surface and releasing them into lysosomes. Indeed, Ndjamen et al. (2014) demonstrated that HSV-1 gE-gI Fc receptor was able to internalize HSV-1 antigen gD through the formation of bipolar bridged complexes in presence of a human anti-gD antibody. The acidic environment of the intracellular sorting endosomes allowed for the dissociation of the Fc region from gE-gI only, while the Fabs portions remained associated with gD. Upon dissociation, the free gE-gI complex was sorted back on the cell surface.

Irrespective of whether they are inserted into the viral particles or escort IgG to degradation, herpesviral Fcγ-binding proteins need to be targeted to the plasma membrane and then enter the endocytic pathway. In infected fibroblasts, prolonged colocalization with EEA1-positive vesicles after internalization, consistent with the biochemical characteristics of gp68 binding to its substrate, led us to hypothesize that bound and internalized antibodies could have a different fate than degradation. Indeed, HCMV gp68 is able to bind the Fc fragment at pH low as 5.6 (Sprague et al., 2008), suggesting that the receptor may not dissociate from the ligand in the endocytic pathway. This hypothesis was strengthened by the work of Manley et al. (2011) demonstrating the ability of HCMV to acquire MSL-109 resistance when virus was grown in presence of this anti-gH neutralizing antibody after infection. In this report, it was shown the incorporation of the antibody into the assembling virions, but the precise localization and mechanism were not addressed. Our work suggests that gp68 is the main responsible of the targeting of MSL-109 to the viral envelope through its Fc moiety while gp34 can internalize IgG but it is dispensable for IgG incorporation into the virion. Ndjamen et al. (2016) identified gp68 as responsible of IgG internalization and degradation. However, in their work, they expressed isolate gp68 in transient transfection in Hela cells, a system that does not fully reproduce the complexity of an infected cell. In our study, we observed a strong overlap between internalized IgGs and EEA1 signals in confocal analysis. This suggests that the majority of IgGs travels to the vAC to be subsequently incorporated in budding virions. However, we cannot exclude that a fraction of internalized antibodies is delivered to the lysosomal compartment *via* gp68 and gp34.

Ideally, all antibodies carrying the IgG Fc region can be incorporated into virions through gp68. In addition, among all anti-HCMV antibodies tested, MSL-109 seems to rely exclusively on gp68 to be transported on the virion. Conversely, monoclonal antibodies 13H11, 1G2, and 4I22, targeting a different epitope of gH, gB, and UL128/130A, respectively, were transported on the viral envelope also independently of the vFcγRs. MSL-109 does not recognize gH on virions and it is thought that the neutralizing epitope forms once gH conformational change occurs to allow viral fusion (Manley et al., 2011). It could be that antibodies able to target their viral glycoprotein on the membrane of infected cells *via* Fab are incorporated into virions following internalization of the complex, but this hypothesis needs further work to be verified. However, to our knowledge, this is the first time that a virus has been found to collect human antibodies from the medium, likely even from human serum *in vivo*, and generate viral particles decorated with these molecules.

In wt viruses, antibodies would be present in two different topologies (a) bound to gp68 with the Fc and exposing the Fab externally and (b) bound *via* Fab to its target protein and projecting the Fc region. However, irrespective of the topology, anti-human antibodies targeting the Fc domain of virus-bound mAbs neutralize infectivity. Manley et al. (2011) also found that the MSL-109 Fc region must be accessible for a productive infection and formulated the hypothesis that an Fc receptor must be involved in the HCMV uptake even if none is known to be present on fibroblasts. We have broadened the spectrum of anti-HCMV antibodies sensitive to this inhibition and shown that this mechanism is gp68 independent for most anti-glycoproteins mAbs. Considering the absence of FcγRs on the host cells, we can speculate that anti-human antibodies binding can create a steric hindrance that impairs proper recognition of the cellular receptor. Anti-human antibodies binding could also cross-link viral particles by bivalent binding, forming aggregates unable to enter the target cells.

We have found that antibody-coating viruses are not protected from neutralization of homologous and heterologous specific Abs, although a clear picture could be obtained only testing a higher number of antibodies and conditions. Thus, why HCMV should acquire specific and nonspecific antibodies on its envelope? We know that at least one neutralizing antibody, the MSL-109, is transported exclusively by gp68 and its incorporation renders the virus resistant to neutralization by the same antibody. Thus, in this specific case, antibody coating represents an immune-evasion escape mechanism. Conversely, antibodies transported independently of gp68 do not provide resistance to homolog neutralization. However, more antibodies must be tested to prove if MSL-109 is the only antibody able to provide protection against itself.

**Figure 9:**
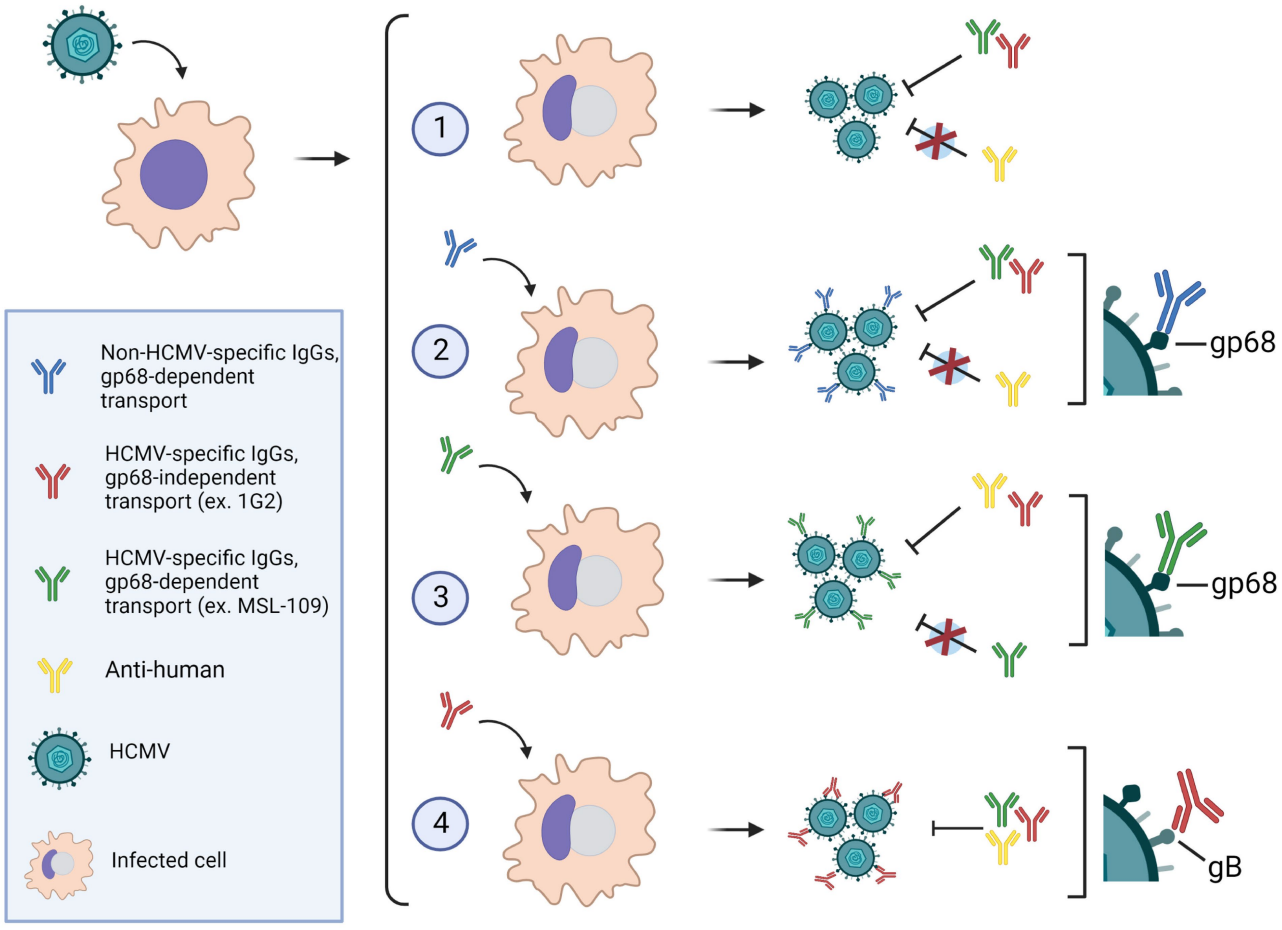
(1) HCMV-infected cells in absence of antibodies, produce viruses that can be neutralized by anti-HCMV specific IgGs, but not by anti-human IgG. (2) In presence of human IgGs, antibodies are internalized *via* gp68 and incorporated on the virion envelope with their Fc portion bound to the vFcR. Virions harboring nonspecific IgGs on their surface can be neutralized by anti-HCMV antibodies but not by anti-human IgGs. (3) The anti-gH neutralizing antibody MSL-109 can also be transported *via* gp68 on the virion surface. In this scenario, the virions are resistant to further neutralization by MSL-109, but sensitive to neutralization with anti-human and other neutralizing anti-HCMV antibodies. (4) Antibodies targeting HCMV envelope glycoproteins can also be incorporated on the viral envelope likely through their Fab moiety bound to the specific glycoprotein. Such virions are susceptible to neutralization by anti-HCMV as well as anti-human antibodies. Image made with BioRender.

In addition, we hypothesized that antibodies exposure on viral surface could raise a gain of function in infection, exploiting the so-called antibody-dependent enhancement (ADE). ADE is caused by binding of non-neutralizing or sub-optimal concentration of neutralizing antibodies to viruses resulting in uptake of multimeric virus-bound IgG in cells expressing Fcγ receptors (FcγRs; Halstead and O’Rourke, 1977). Although ADE has been evoked in the exacerbation of several viral diseases, *in vivo* robust evidence of the direct implication of FcγRs-dependent internalization in causing severe disease has been obtained only for dengue virus (DENV) (Katzelnick et al., 2017; Bournazos et al., 2020; Farouq et al., 2022) and not found with vaccines against other herpesviruses such as VZV or HSV. The DENV entry into phagocytic cells, through FcγRs, allow the IgGs-opsonized virus to reach the endosomal low pH milieu promoting conformational changes of the fusion protein and subsequent infection (Pierson and Kielian, 2013). We have shown that HCMV can capture specific antibodies through their Fab domain and nonspecific IgGs through their Fc. The first modality would provide a “canonical” ADE mechanism but also the characteristic of the gp68 binding to IgGs would allow engagement to host FcγRs. Indeed, Sprague et al. (2008) found that the gp68-binding region on the Fc differs from those of host FcγRs. More recently, Kolb et al. infected cells with wt virus and mutants expressing individually gp34 or gp68 in presence of opsonizing IgGs to analyze the interference to human FcγRs recognition to the Fc region. The authors found that individual expression of gp34 or gp68 reduced but not abolished IgGs recognition by host FcγRIIIA, FcγRIIA, and FcγRsIIB/C, while FcγRI binding was almost unaffected (Kolb et al., 2021). The contemporary presence of both gp34 and gp68, however, completely antagonized all host FcγRs. These data led to an elegant model where gp68 and gp34 work in cooperation by simultaneously blocking and internalizing immune complexes from the infected cells plasma membrane and therefore antagonize the activation of NK cells activation (Kolb et al., 2021). Such mechanism cannot be performed by gp34 and gp68 when present on the viral envelope, thus it is obvious to speculate that, on virions, vFcR must exert a different function. Our data showed that the gp68 protein carries most of the IgGs on virions and it is tempting to speculate that most of the complexes gp68-IgGs might be free of gp34. In that case, gp68-bound IgGs could still recognize host FcγRs and mediate virus uptake. Findings reported in this work are summarized in Figure 9. This scenario pictures the ability of the virus to capture circulating antibodies in two distinct manners suggesting that HCMV owns a particular mechanism of immune escape still to be deciphered.

For instance, in a HCMV infection *in vivo* in naïve individuals, binding nonspecific human IgGs would provide an additional weapon for the virus to infect cells of the innate immune system. In this scenario, monocytes would be the target population due to their role in dissemination and as reservoir of latent viruses (Collins-McMillen et al., 2018). Upon productive HCMV infection of monocytes, various signaling pathways are induced, among which a key role is played by EGFR-mediated induction of PI(3)K, Akt, N-WASP, NF-κB, and Sp-1, promoting survival and differentiation (reviewed in Lee et al., 2021). Monocytes express the activatory receptors, FcγRI, FcγRIIa, and FcγRIIIa, the latter only in 10% of the monocytes, and the inhibitory receptor, FcγRIIb. Once engaged by opsonized pathogens, the activatory receptors trigger pathways leading to the activation of “*de facto*” the same effectors (Bournazos et al., 2020). Altogether, we consider this hypothesized immune escape mechanism worthy of further investigations.

Fcγ-binding proteins carried by viral particles are present on other species, but this peculiar function has never been described. The major VZV envelope glycoprotein gE is an essential viral protein that endows an Fc receptor activity linked to other functions including binding to receptors (Litwin et al., 1992; Chen et al., 2004; Maresova et al., 2005; Li et al., 2006). Remarkably, adjuvanted recombinant VZV gE is protective as single antigen vaccine (Heineman et al., 2019). The HSV-1 gE envelope glycoprotein is a Fcγ-binding protein classified as non-essential for *in vitro* growth but crucial for cell-to-cell spread. Furthermore, gE null or mutated in the Fcγ-binding domain impaired neurovirulence *in vivo* (Johnson et al., 1988; Dingwell et al., 1994; Saldanha et al., 2000). Mostly associated with gI, the complex undergoes endocytosis upon IgG binding at the plasma membrane (Ndjamen et al., 2014). All HCMV vFcγRs undergo internalization and follow the same route to viral particles and it would be interesting to verify if VZV or other herpesviruses carry envelope IgGs. Association of HSV-1 gE with the IgG is pH sensitive and it has been inferred that IgGs would be released during acidification of the endocytic pathway (Sprague et al., 2004). However, it is not clear at which stage of the endosome maturation sorting of viral protein to vAC occurs and it could also be that part of the complexes destined to viral particles never reach pH close to 6.0 and is not released.

Due to the lack of an animal model for HCMV, it would be very challenging to assess the importance of vFcγRs *in vivo*, however further functional characterization of these mechanisms will provide a better understanding of this immune escape mechanism and better define the role of this class of proteins in HCMV immune-evasion and pathogenesis.

## Data availability statement

The raw data supporting the conclusions of this article will be made available by the authors, without undue reservation.

## Author contributions

GV, SP, RF, SC, GM, DA, and MC performed the experiments. EF, DM, MC, and MM were involved in the conception and design of the study. GV, SP, RF, SC, and GM acquired the data. GV, MC, and MM analyzed and interpreted the results. At the time of this project, GV was a PhD student at University of Bologna. All authors were involved in drafting the manuscript or revising it critically for important intellectual content. All authors contributed to the article and approved the submitted version.

## Funding

This work was sponsored by GlaxoSmithKline Biologicals SA and there was no additional funding. GSK took responsibility for all costs incurred in publishing.

## Conflict of interest

EF and DM are employed by GSK. DM and EF report ownership of GSK shares and/or restricted GSK shares. GV, SC, DA, and MC were PhD students sponsored by GSK vaccines. SP, RF, and GM were graduate students in an internship in GSK. MM is an employee of the University of Naples Federico II with a consultancy contract with GSK.

The authors declare that this study was sponsored by GlaxoSmithKline Biologicals SA. The sponsor had the following involvement in the study: study design, collection, analysis, interpretation of data, writing of this article and decision to submit it for publication.

## Publisher’s note

All claims expressed in this article are solely those of the authors and do not necessarily represent those of their affiliated organizations, or those of the publisher, the editors and the reviewers. Any product that may be evaluated in this article, or claim that may be made by its manufacturer, is not guaranteed or endorsed by the publisher.
